# Effects of chilling on the photosynthetic performance of the CAM orchid *Phalaenopsis*

**DOI:** 10.3389/fpls.2022.981581

**Published:** 2022-11-25

**Authors:** Stijn Daems, Nathalie Ceusters, Roland Valcke, Johan Ceusters

**Affiliations:** ^1^ Research Group for Sustainable Crop Production & Protection, Division of Crop Biotechnics, Department of Biosystems, KU Leuven, Geel, Belgium; ^2^ KU Leuven Plant Institute (LPI), KU Leuven, Leuven, Belgium; ^3^ Molecular and Physical Plant Physiology, UHasselt, Diepenbeek, Belgium; ^4^ Centre for Environmental Sciences, Environmental Biology, UHasselt, Diepenbeek, Belgium

**Keywords:** chlorophyll fluorescence, OJIP, crassulacean acid metabolism, photosynthesis, *Phalaenopsis*, chilling

## Abstract

**Introduction:**

Crassulacean acid metabolism (CAM) is one of the three main metabolic adaptations for CO_2_ fixation found in plants. A striking feature for these plants is nocturnal carbon fixation and diurnal decarboxylation of malic acid to feed Rubisco with CO_2_ behind closed stomata, thereby saving considerable amounts of water. Compared to the effects of high temperatures, drought, and light, much less information is available about the effects of chilling temperatures on CAM plants. In addition a lot of CAM ornamentals are grown in heated greenhouses, urging for a deeper understanding about the physiological responses to chilling in order to increase sustainability in the horticultural sector.

**Methods:**

The present study focuses on the impact of chilling temperatures (10°C) for 3 weeks on the photosynthetic performance of the obligate CAM orchid *Phalaenopsis* ‘Edessa’. Detailed assessments of the light reactions were performed by analyzing chlorophyll *a* fluorescence induction (OJIP) parameters and the carbon fixation reactions by measuring diel leaf gas exchange and diel metabolite patterns.

**Results and Discussion:**

Results showed that chilling already affected the light reactions after 24h. Whilst the potential efficiency of photosystem II (PSII) (F_v_/F_m_) was not yet influenced, a massive decrease in the performance index (PI_abs_) was noticed. This decrease did not depict an overall downregulation of PSII related energy fluxes since energy absorption and dissipation remained uninfluenced whilst the trapped energy and reduction flux were upregulated. This might point to the presence of short-term adaptation mechanisms to chilling stress. However, in the longer term the electron transport chain from PSII to PSI was affected, impacting both ATP and NADPH provision. To avoid over-excitation and photodamage plants showed a massive increase in thermal dissipation. These considerations are also in line with carbon fixation data showing initial signs of cold adaptation by achieving comparable Rubisco activity compared to unstressed plants but increasing daytime stomatal opening in order to capture a higher proportion of CO_2_ during daytime. However, in accordance with the light reactions data, Rubisco activity declined and stomatal conductance and CO_2_ uptake diminished to near zero levels after 3 weeks, indicating that plants were not successful in cold acclimation on the longer term.

## Introduction

Plants thrive in a wide range of natural environments characterized by ever-changing weather conditions, bringing about a multiplicity of stresses. Consequently, plants have evolved an extensive arsenal of ecological adaptations. Crassulacean acid metabolism (CAM) is a well-known photosynthetic adaptation to optimize water use efficiency (WUE) by taking up atmospheric CO_2_ predominantly during the night when evapotranspiration is low (Borland et al., 2011; Winter, 2019). This important trait enables CAM plants to survive in water-limited environments such as semi-arid deserts or seasonally dry forests. Very recently, a large interest has arisen to engineer the CAM pathway into important non-CAM food, feed, and bioenergy crops grown in abandoned, marginal, semi-arid, or degraded agricultural lands (DePaoli et al., 2014; Yang et al., 2015; Lim et al., 2019). In general, diel CAM can be divided into four specific phases of gas exchange, which are also useful to describe photosynthetic performance. Nocturnal opening of the stomata allows fixation of atmospheric CO_2_ (Phase I) by concerted action of the enzymes carbonic anhydrase and phosphoenolpyruvate carboxylase (PEPC). The latter requires a 3-C substrate i.e. phosphoenolpyruvate (PEP) which is provided by the glycolytic breakdown of carbohydrates. The final 4-C product, malate, is stored in the central vacuole. During daytime, gas exchange with the atmosphere gets obstructed by stomatal closure (Phase III), allowing great water savings and improved WUE by reducing water losses *via* transpiration. Malic acid from the vacuole is then released and decarboxylated, feeding CO_2_ to ribulose-1,5-bisphosphate carboxylase-oxygenase (Rubisco) for assimilation in the Calvin-Benson cycle. Decarboxylation is catalyzed by either NAD(P)-malic enzyme (ME) or phosphoenolpyruvate carboxykinase (PEPCK), depending on the CAM species considered. At either the start (Phase II) or end of the day (Phase IV), stomata are open to allow atmospheric CO_2_ sequestration by combined action of both PEPC and Rubisco (Osmond, 1978; Borland et al., 2011). The four CAM phases show great plasticity in terms of duration and magnitude which is a key factor to ameliorate carbon gain and water use under changing environmental conditions (Lin and Hsu, 2004; Ceusters et al., 2011; Tay et al., 2019; Ceusters et al., 2021).

Global climate change is one of the major challenges for our society. Projections of various model experiments on future climate change scenarios clearly indicate a rise in mean global temperature in the longer term (Arnell et al., 2019; Chiang et al., 2021; Zandalinas et al., 2021). Model predictions from Johnson et al. (2018) indicate that summer warm extremes will increase more in frequency than winter cold extremes. However, despite this predicted global warming scenario for the next decades, analyses of climate model simulations and observations also uncover that periods of cold are likely to persist in intensity and duration, but not in frequency, across each land-continent (Kodra et al., 2011). Most tropical plants are cold-sensitive and can already encounter physiological problems at temperatures just below 15°C (Chen, 1994). Potential cooling damage depends on the chilling rate, exposure time, and other associated stresses (Buchanan et al, 2015). Chilling injury can already occur within a few hours when exposed to cool temperature conditions and can compromise essentially all key components of photosynthesis including thylakoid electron transport, control of stomatal conductance, carbon reduction, and carbohydrate partitioning (McConnell and Sheehan, 1978; Allen and Ort, 2001; Lundmark et al., 2006).

The response of chlorophyll fluorescence parameters to chilling temperatures has already been evaluated. In general, a significant decrease in the potential efficiency of photosystem II (PSII) (F_v_/F_m_) during chilling has been observed in multiple C_3_ plants, indicating a photo-inhibitory effect (Agati et al., 1996; Lin et al., 2007; Zhang et al., 2010; Hou et al., 2016; Bhandari et al., 2018). Photoinhibition consists of an arsenal of complex mechanisms aimed to protect the photosynthetic apparatus from excess light energy and can be depicted as a balancing process between the rate of photodamage to PSII and the rate of its repair. In Greer et al. (1986) already observed photoinhibition in *Phaseolus vulgaris* at 5°C and 140 µmol m^-2^ s^-1^ and proposed that low temperatures mainly suppressed the PSII repair mechanism. More specifically, a diminished fixation of CO_2_ in the Calvin-Benson cycle, due to environmental stresses (i.e. low temperatures), limits the consumption of ATP and NADPH. This causes a concomitant drop in the level of NADP^+^ which is the major acceptor of electrons in PSI. Eventually, this NADP^+^ depletion accelerates transport of electrons from PSI to molecular oxygen with the resultant accumulation of reactive oxygen species (ROS), which are supposed to inhibit synthesis of the D1 protein and other proteins of PSII required for the photodamage-repair cycle (Allakhverdiev and Murata, 2004; Murata et al., 2007). In addition, low temperatures might also destabilize the translation machinery directly, attenuating repair capacity (Takahashi and Murata, 2008). Also for CAM plants recent observations in *Aloe vera* showed a low temperature tolerance with regard to several important parameters of the Chl fluorescence transient (OJIP) such as F_v_/F_m_ and PI_abs_ in plants exposed to chilling stress (2°C). The latter parameter quantifies the overall functionality of the electron flow through PSII and is highlighted as a sensitive parameter for plant homeostasis (Rapacz, 2007; Živčák et al., 2008). Moreover, lower values of photosynthetic electron transport chain components including the electron transport flux (φE_o_) and the inferred oxygen-evolving complex activity (F_v_/F_o_) were also observed (Habibi, 2019). A reduction in the content of photosynthetic pigments induced by cold may also further contribute to a decrease in photosynthesis (Haldimann, 1999).

Besides the performance of energy (ATP) and reducing power (NADPH) supply *via* the light reactions, gas exchange parameters such as stomatal conductance (g_s_) and internal leaf CO_2_ concentration (C_i_) in relation to enzyme activities are also pivotal parameters to consider when evaluating effects of chilling. First, closing of stomata after a chill can be a direct low temperature effect on functioning of guard cells. Cold sensitive plants have less capacity to increase Ca^2+^ uptake by guard cells and therefore closing of stomata is delayed (Wilkinson et al., 2001; Hussain et al., 2018). Second, a chill-induced reduction of Rubisco activity could lead to a rise in C_i_, which in turn induces stomatal closure (Allen and Ort, 2001). Activities of key enzymes involved in carbon fixation in CAM plants have indeed been reported to decline when exposed to chilling possibly causing lower photosynthetic rates. The rate of diurnal CO_2_ fixation is determined by the rate of CO_2_ liberation by ME or PEPCK, CO_2_ assimilation rate by Rubisco, and/or photosynthetic electron transport in the thylakoid membranes, whereas in the dark, it is determined by the rate of CO_2_ uptake by PEPC. According to Cen and Sage (2005), Rubisco activity *in vitro* declined exponentially from 45°C to 5°C. More specifically, Rubisco activity at 10°C was approximately 10 times lower compared to 30°C in sweet potato (*Ipomoea batatas*) leaves. Chilling can also damage Rubisco itself or affect the redox regulation of the larger Rubisco activase isoform (Byrd et al., 1995; Kingston-Smith et al., 1997; Zhang and Portis, 1999). Yamori et al., (2005; 2011; 2014) found that C_3_ plants grown at low temperatures possess higher contents of different enzymes involved in the photosynthetic carbon reduction cycle, such as Rubisco, sedoheptulose-1,7-bisphosphatase (SBPase), and stromal fructose-1,6-bisphosphatase (FBPase). In addition, enzymes involved in sucrose synthesis such as sucrose phosphate synthase (SPS) and cytosolic FBPase also show higher contents in plants grown under low temperatures (Hurry et al., 1994; Hurry et al., 1995; Yamori et al., 2014). As such, reduced intrinsic enzyme activities at low temperatures will be compensated by larger amounts of these enzymes. Moreover, expression of cold stable isozymes with optimized performance at low temperatures bring about an extra compensation for reduced enzyme activity (Yamori et al., 2014).

Dong and Beckles (2019) postulated that localized starch degradation into sugars is vital for a proper cold stress response. The sugars that are produced provide osmoprotection and rapid energy supplies for protective functions (i.e. respiration, substrates needed for the biosynthesis of protective proteins and compounds, and basic metabolism for survival). Gene expression and functional studies have indicated that several β-amylase (BMY) isoforms are activated upon cold (Fowler and Thomashow, 2002; Jung et al., 2003; Seki et al., 2003). An excessive amount of source sugars may protect sensitive membranes and proteins from dehydration due to cold, but they are hardly accessible for growth, and may thus instead eventually inhibit photosynthesis (Lemoine et al., 2013). Carvalho et al. (2013) explored the effects of chilling stress on the bromeliad *Nidularium minutum*. A higher glucose content in the leaves of this bromeliad was observed when maintained at 10°C for six months compared to those grown at 25°C, thus indicating a cryoprotection function for this carbohydrate. Wattana (2003) studied the effects of chilling stress on raffinose family oligosaccharides (RFOs) (derivatives of sucrose to which one or two galactosyl units are added) (ElSayed et al., 2014) metabolism in *Plectranthus hadiensis* L., a CAM species of the Lamiaceae which uses RFOs for translocation and storage in the mesophyll cells. RFO concentrations increased and remained greater in chilled plants throughout the 18 days chilling treatment. In addition, galactinol synthase (GS) activities increased in the chilled plants compared to the controls. These results suggest that a mesophyllic pool of RFOs can also play a key role in cold tolerance.

Carbon partitioning within sources and sinks, and its transport between them, is also affected by temperature which can alter source/sink relationships (Lemoine et al., 2013). Overall, at a relatively low temperature the production of assimilates continues but the translocation and uptake fall to lower levels causing assimilates to accumulate. Accumulation of non-structural carbohydrates in both sources and sinks is a typical phenomenon observed at low temperatures. This is partly due to growth being more sensitive than photosynthesis to reductions in temperatures, and partially due to the distinctive sensitivity that enzymes involved in carbohydrate metabolism show to temperature. Transportation in the phloem is reduced by low temperature, due partly to viscosity and partly, possibly, to displacement of the contents of sieve elements (Farrar, 1988). From all of the above it can be deduced that translocation rates of photoassimilates are also dramatically influenced by chilling conditions. Toki et al. (1978) and Miao et al. (2007) reported a low translocation rate from source to sink in cucumber. In mature leaves, photoassimilates were exported within 2 h at 20°C and 4 h at 16°C. At 10°C, translocation was even stronger obstructed with only half of the assimilates exported.

In contrast to the effects of high temperatures, drought, and light, much less information is known about the effects of chilling temperatures on CAM photosynthesis. The purpose of the present work was to gain more insights into the effects of chilling temperatures (10°C) on photosynthetic performance in tropical CAM plants by consideration of different aspects of photosynthesis i.e. the light reactions and carbon fixation. These thermophilic plants offer the opportunity to investigate the effects of chilling on photosynthetic processes undisguised by innumerable protective responses observed in chilling tolerant species (Allen and Ort, 2001). Therefore, a comprehensive study was performed integrating determinations of fast chlorophyll *a* fluorescence, pigment contents, Rubisco activity, diel leaf gas exchange patterns, and diel metabolite dynamics (e.g. malic acid, starch and soluble sugars) in leaves of the CAM orchid *Phalaenopsis* ‘Edessa’. The findings in this study will not only advance our understanding of chilling sensitivity in tropical CAM plants but will also be valuable for the horticultural sector. Since a lot of CAM ornamentals such as *Phalaenopsis*, *Kalanchoë*, many other orchids and bromeliads are grown in heated greenhouses, deeper insights in physiological responses to chilling might enhance efforts to increase sustainability in the sector.

## Materials and methods

### Plant material and sampling

*Phalaenopsis* ‘Edessa’ is an obligate starch-storing CAM plant and belongs to the family of the Orchidaceae. Vegetative plants (plants that have not yet flowered) of 16 weeks old were cultivated in a growth room with a constant day/night temperature of 10°C, a relative humidity (RH) of 75% and a 12-h photoperiod (zeitgeber time ZT0-ZT12) with photosynthetic photon flux density (PPFD) of 100 µmol m^-2^ s^-1^. Watering was performed twice a week; once with a nutrient solution Peters 20N-8.7P-16.6K of 1 mS cm^-1^ and once with water. Control plants were kept at 28°C day and night. Other relevant parameters such as RH, photoperiod, PPFD, and watering were kept comparable to the conditions as described above. After 0 (control), 1, 8, 15 and 22 days, leaf samples (n = 5) were taken from the upper one-third of young fully expanded source leaves during the diel cycle starting from 08.00 h (ZT0) every 2 h until 08.00 h the next morning (ZT24). The samples from 08.00 h to 18.00 h (ZT0/ZT24 to ZT10) were taken when the lights were turned on whilst the samples taken from 20.00 h to 06.00 h (ZT12 to ZT22) were taken in the dark under green safety light. All samples were immediately frozen in liquid nitrogen, powdered and stored at -80°C until analysis.

### Chlorophyll *a* fluorescence and JIP-test

Chlorophyll *a* fluorescence measurements were carried out by means of a Handy PEA fluorometer (Plant Efficiency Analyser, *Hansatech*, King’s Lynn, United Kingdom) and were taken on the adaxial side, always on the left side from the main vein, of young fully expanded leaves (n = 15 plants). Measurements were performed using saturating red light pulses of 3 000 µmol m^-2^ s^-1^, peak wavelength at 650 nm, pulse duration of 1 s, and fixed gain (1.2x). Induction curve analysis (Handy PEA software V1.10) allowed to evaluate the effectiveness of fluorescence saturation during measurements. Before carrying out measurements, leaves were always allowed to dark adapt for 20 min using light-withholding clips (*Hansatech*). The light level of the saturating pulse and minimal dark period had experimentally been determined for *Phalaenopsis* ‘Edessa’ beforehand in order to obtain true values for the chlorophyll *a* fluorescence parameters. During the experiment chlorophyll measurements were taken weekly during one month at 08.00 h (Phase II). All chlorophyll fluorescence data were assembled at 08.00 h because additional measurements at 12.00 h (Phase III) revealed no significant differences for these parameters in the different CAM phases (Ceusters et al., 2019).

The measured fast chlorophyll fluorescence induction curves (F_0_ to F_m_) were analyzed by the JIP test, which is based on the theory of energy fluxes in the photosynthetic apparatus (Strasser et al., 2000; Stirbet and Govindjee, 2011). When receiving light, part of the flux of photons absorbed by PSII antenna pigments (ABS) is dissipated (DI), mainly as heat and another part is converted to redox energy by reducing the electron acceptor Q_A_ (TR). This electron acceptor is then reoxidized creating an electron transport (ET) until the reduction of the end electron acceptors at the PSI electron acceptor side (RE). This stepwise flow of energy through PSII can also be expressed per reaction center (RC), defined as following specific energy fluxes: ABS/RC, Di_0_/RC, Tr_0_/RC, Et_0_/RC, and Re_0_/RC. All these specific fluxes refer to time zero, i.e., the onset of fluorescence inductions. Since fluorescence induction data may be affected by the existence of PSII excitonic connectivity, i.e., transfer of excitation energy from a closed PSII RC to an open (active) PSII RC (Stirbet, 2013), it was interesting to take this process into account (Ceusters et al., 2019). Based on Zivcak et al. (2014) the curvature constant (C) of the initial phase of the O-J curve (from 0.05 to 2 ms) was used to correct the values of specific energy fluxes for connectivity, i.e., multiplying the specific energy flux values by 1 + C (Ceusters et al., 2019).

In a logarithmic time scale, fast chlorophyll fluorescence induction curves have a typical shape which show the steps O, J, I, P (Strasser and Strasser, 1995; Srivastava et al., 1999; Strasser et al., 2000; Strasser et al., 2004; Strasser et al., 2010), making it possible to collect following cardinal points: maximal fluorescence intensity (F_m_, when all RCs are closed), minimum fluorescence intensity (F_0_, when all RCs are open), fluorescence intensity at 2 and 30 ms (F_J_ and F_I_, respectively) and at 50 and 300 µs (F_50 µs_ and F_300 µs_). These primary data were used to calculate chlorophyll fluorescence parameters describing maximum quantum efficiency of PSII (F_v_/F_m_), performance index (PI_abs_) and the afore mentioned specific energy fluxes (Chondrogiannis and Grammatikopoulos, 2016). Definitions and equations of the measured and calculated JIP parameters are described in Ceusters et al. (2019).

Relative variable fluorescences at time t, at the J-step, and the I-step (i.e., V_t_, V_J_, V_I_, respectively) were calculated using the following equations (Strasser et al., 2004; Li et al., 2010):


(1) $$V_{t}\;\left(FvF\right)=\left(F_{t}-F_{o}\right)/\left(F_{M}-F_{o}\right)$$

(2) $$V_{J}=\left(F_{J}-F_{o}\right)/\left(F_{M}-F_{o}\right)$$

(3) $$V_{I}=\left(F_{I}-F_{o}\right)/\left(F_{M}-F_{o}\right)$$

(4) $$\text{ Δ}V_{t}\;=\;V_{t\left(chilling\right)}-\;V_{t\left(control\right)}$$

where F_J_ is fluorescence intensity at 2 ms and F_I_ is the fluorescence intensity at 30 ms. To further elucidate the effect of chilling on *Phalaenopsis* ‘Edessa’ PSII, several functional parameters were calculated from the JIP-test. For visualization of the L-band (150 µs), fluorescence data were normalized between O (50 µs) and K (300 µs), as W_OK_ = (F_t_ – F_0_)/(F_K_ – F_0_) and plotted as difference kinetics ΔW_OK_ = W_OK(chilling)_ – W_OK(control)_ (Yusuf et al., 2010). For visualization of the K-band (300 µs), fluorescence data were normalized between O and J (2 ms) steps, as W_OJ_ = (F_t_ – F_0_)/(F_J_ – F_0_) and plotted as difference kinetics ΔW_OJ_ = W_OJ(chilling)_ – W_OJ(control)_ (Yusuf et al., 2010). Fluorescence data were additionally normalized between O and I (30 ms), as W_OI_ = (F_t_ – F_0_)/(F_I_ – F_0_) and plotted as difference kinetics ΔW_OI_ = W_OI(chilling)_ – W_OI(control)_ (Yusuf et al., 2010).

The F_I_, F_J_, F_K_, F_M_, and F_O_ represent fluorescence at I-step, J-step, and K-step, dark-adapted maximum fluorescence, and dark-adapted minimum fluorescence, respectively. ΔV_J_, ΔV_I_, ΔW_OK_ and ΔW_OJ_ represent the J-band, I-band, L-band, and K-band, respectively, and are associated with the accumulation of Q_A_^-^ (Strasser et al., 2004), the fraction of Q_B_-non-reducing PSII RCs (Li et al., 2010), energetic connectivity of antennae to PSII RC units (Yusuf et al., 2010), and the activity of OEC of PSII donor side, respectively (Strasser, 1997; Liang et al., 2019). ΔW_OI_ provides information on blockage of PSII electrons up to plastoquinone (PQ) (Yusuf et al., 2010).

### Determination of chlorophyll and carotenoid content

For the calculation of leaf chlorophyll and carotenoid contents, plant pigments were extracted by immersing leaf material in N,N-dimethylformamide (DMFA) at room temperature for 72 h in darkness (n = 5 plants). The supernatant was used to determine absorbance at 647 nm (A_647_), 664 nm (A_664_) and 480 nm (A_480_) (Genesys 10S UV-VIS, Thermo Fisher Scientific, United States). These data were used to calculate the content of chlorophyll *a*, chlorophyll *b*, total chlorophyll and total carotenoids by means of the empirical formulas: C_a_ = 11.65 A_664_ – 2.69 A_647_; C_b_ = 20.81 A_647_ – 4.53 A_664_; C = C_a_ + C_b_; C_x+c_ = (1000 A_480_ – 0.89 C_a_ – 52.02 C_b_)/245 respectively (Moran and Porath, 1980; Porra et al., 1989; Wellburn, 1994).

### Enzyme activity of Rubisco

The extraction and assay of Rubisco were based on the method described by Borland et al. (1998). About 100 mg powdered leaf tissue was homogenized in 300 µL extraction buffer at 4°C containing: 400 mM HEPES-KOH (pH 7.5), 5 mM EGTA, 5 mM MgCl_2_, 2% (w/v) polyethylene glycol (PEG) 20 000, 14 mM β-mercaptoethanol, 16 mg polyvinylpolypyrrolidone (PVPP), and 1 mM PMSF. The homogenate was centrifuged at 16 200 *g* for 2 min at 4°C. The initial activity of Rubisco was assayed in a reaction mix containing: 100 mM Bicine-KOH (pH 8.0), 25 mM NaHCO_3_, 20 mM MgCl_2_, 3.5 mM ATP, 3.5 mM P-creatine, 0.25 mM NADH, 5 units 3-phosphoglyceric phosphokinase, 5 units glyceraldehyde 3-phosphate dehydrogenase, and 5 units creatine phosphokinase. After preincubation for 10 min at 25°C, the reaction was initiated by the addition of RuBP to a final concentration of 0.5 mM and change in absorbance at 340 nm was measured for 4 min at 25°C. Preliminary experiments confirmed a linear decrease of NADH for at least 6 min.

### Gas exchange measurements

Gas exchange parameters (net CO_2_ uptake, stomatal conductance, and transpiration) were measured weekly for 4 weeks [day 0 (control), day 8, 15, and 22] on the youngest fully expanded leaves, using a LCi Portable Photosynthesis System (ADC BioScientific Ltd., United Kingdom). The top part of the leaf was enclosed in a broad lead chamber (6.25 cm^2^) and the incoming air was passed through a 20-l bottle to buffer short-term fluctuations in the CO_2_ concentration. Gas exchange data were collected over the diel cycle with measurements obtained at 15-min intervals (n = 3 plants). By integrating specific areas under the gas exchange curves [CO_2_ and transpiration (H_2_O)], net gas exchange was calculated for day and night as well as total net gas exchange during the 24-h period.

### Chemical analyses of metabolites

Extraction for measurements of malic acid and starch was performed as described by Chen et al. (2002), but with modifications. Approximately 180 mg of powdered tissue was mixed with 450 µl of ice-cold 4% (v/v) HClO_4_. The mixture was allowed to thaw slowly on ice for 30 min. The resulting suspension was then centrifuged at 4°C for 10 min at 16 200 *g*. The insoluble residue from the perchloric acid extraction was used to determine starch content spectrophotometrically at 340 nm as glucose equivalents (Genesys 10S UV-VIS, Thermo Fisher Scientific, United States), following digestion with a mix of amyloglucosidase (EC 3.2.1.3) and α-amylase (EC 3.2.1.1). The analyses were conducted as earlier described by Ceusters et al. (2008). The supernatant from the HClO_4_ extraction was neutralized at 4°C with 5 M K_2_CO_3_, and the resulting potassium perchlorate precipitate was removed by 10 min centrifugation at 16 200 *g* and 4°C. Five mg activated charcoal was added to the supernatant, and after 15 min at 4°C, removed by new centrifugation. The supernatant was used for measurement of malic acid. Malic acid was measured in a 500-µl reaction mixture (EnzytecTM code n° E1215) containing: glycylglycinebuffer, NAD, glutamate oxaloacetate transaminase (GOT, EC 2.6.1.1). Analysis was performed spectrophotometrically by determining the change in absorbance at 340 nm after adding L-malate dehydrogenase (L-MDH, EC 1.1.1.37).

Soluble sugars (sucrose, glucose, and fructose) were extracted using hot water (80°C), as described by Tarkowski et al. (2019), and quantified by high performance anion exchange chromatography with pulsed amperometric detection, as described by Verspreet et al. (2013). Metabolites were only compared at day 0 (control) and after 3 weeks (day 22) of chilling.

### Data analyses

Where appropriate, data were analyzed using the statistical software package IBM SPSS Statistics V23. Before carrying out statistical tests, normality of the data was checked by means of the Kolmogorov-Smirnoff statistic (P > 0.05). Throughout the manuscript means are compared by analysis of variance (ANOVA) followed by Tukey’s HSD *post-hoc* test (α = 0.05) except for comparisons between 2 groups by an independent sample t-test (α = 0.05). All replicates considered in our study were independent biological replicates originating from different plants.

## Results

### Chlorophyll *a* fluorescence parameters and OJIP curves

**Figure 1:**
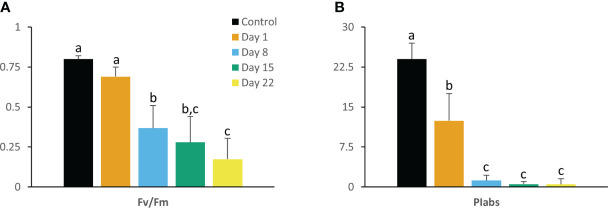
Maximum quantum efficiency of PSII (F_v_/F_m_) **(A)** and performance index (PI_abs_) **(B)** measured at 08.00 h in young fully expanded leaves of *Phalaenopsis* ‘Edessa’ at 0 (control), 1, 8, 15 and 22 days after exposure to chilling conditions (10°C). Data are means ± SE (n = 15 plants). Values were compared among the different time points according to Tukey’s HSD test at p < 0.05 marked by different letters.

**Figure 2:**
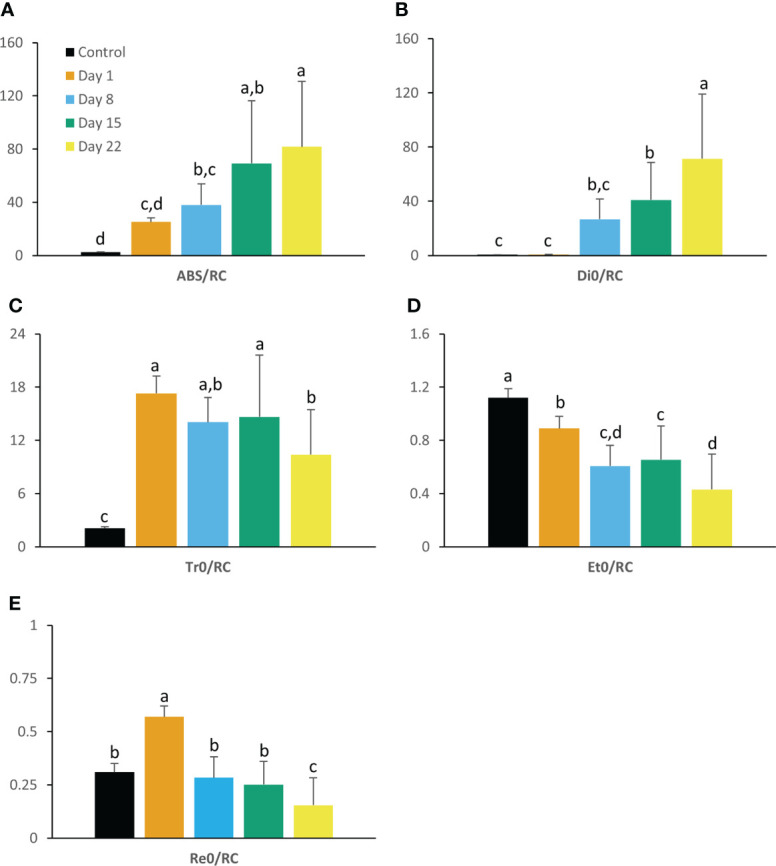
Absorption per active PSII reaction center (ABS/RC) **(A)**, dissipation (Di_0_/RC) **(B)**, trapping (Tr_0_/RC) **(C)**, electron transport (Et_0_/RC) **(D)** and reduction flux (Re_0_/RC) **(E)** measured at 08.00 h in young fully expanded leaves of *Phalaenopsis* ‘Edessa’ at 0 (control), 1, 8, 15 and 22 days after exposure to chilling conditions (10°C). Data are means ± SE (n = 15 plants). Values were compared among the different time points according to Tukey’s HSD test at p < 0.05 marked by different letters.

Regarding the progression of chlorophyll fluorescence parameters with correction for connectivity [i.e. multiplying the specific flux values by 1 + C (Zivcak et al., 2014)], the maximum quantum efficiency of PSII photochemistry (F_v_/F_m_) and the performance index for absorption (PI_abs_) were largely affected by chilling (10°C) (**Figure 1**). Values of F_v_/F_m_ decreased gradually as the experiment progressed in contrast to values of PI_abs_ which already reduced drastically (P < 0.05) after 1 day followed by a further strong decline at day 8 (P < 0.05) to near zero levels. Cold stress also affected the specific energy fluxes within and related to PSII. A gradual increase over 22 days was noticed for photon absorption (ABS/RC) (**Figure 2A**) whilst an augmented activity of energy dissipation (Di_0_/RC) was noticed especially at the end of the chilling period (days 15 and 22) (**Figure 2B**). Already after 1 day significant (P < 0.05) increases were noticed for both the electron trapping efficiency (Tr_0_/RC) (**Figure 2C**) and the flow of electrons further than PSII (Re_0_/RC) (**Figure 2E**). Whilst the latter showed a gradual decrease from day 8 to 22, the first remained rather stable and only started to decline at day 22. Electron transport within the reaction center (Et_0_/RC) gradually diminished (P < 0.05) throughout the whole experiment (**Figure 2D**).

**Figure 3:**
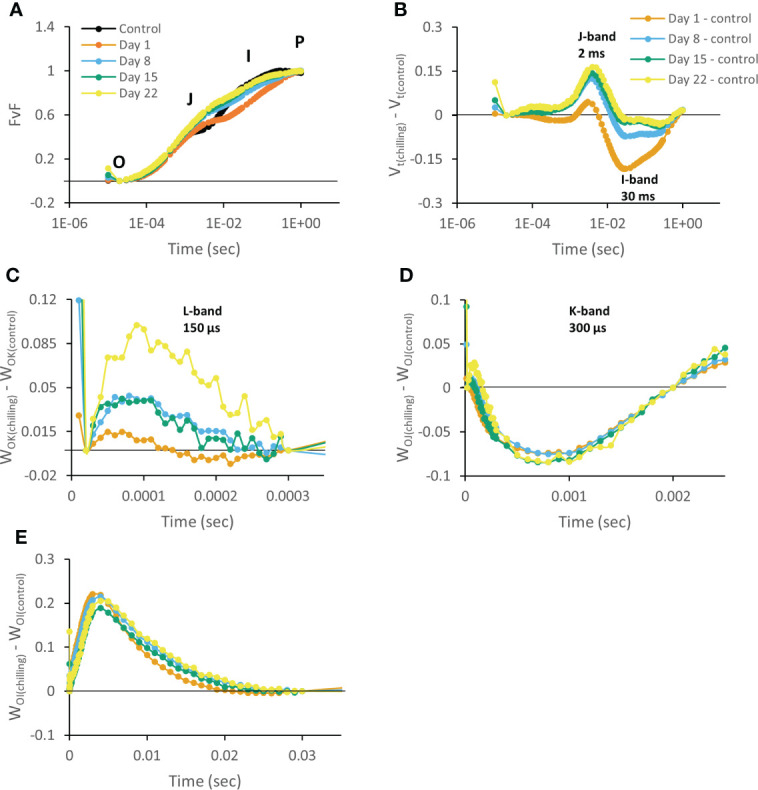
Chlorophyll *a* fluorescence OJIP transient curves measured at 08.00 h in young fully expanded leaves of *Phalaenopsis* ‘Edessa’ at 0 (control), 1, 8, 15 and 22 days after exposure to chilling conditions (10°C). Transients were double normalized between O and P steps: V_t_ (FvF) = (F_t_ – F_0_)/(F_M_ – F_0_) and plotted on a logarithmic time scale **(A)**. Plotted relative variable fluorescence V_t(chilling)_ – V_t(control)_ for visualization of J-band (ΔV_J_) and I-band amplitudes (ΔV_I_) on a logarithmic time scale **(B)**. Changes in O-K phase relative variable fluorescence intensity (ΔW_OK_, L-band) **(C)**, O-J phase relative variable fluorescence intensity (ΔW_OJ_, K-band) **(D)** and in O-I phase relative variable fluorescence intensity (ΔW_OI_) **(E)**. Averaged transients from each time point are illustrated (n = 15 plants). The zero line represents the control group in B, C, D, and E.

The fluorescent transient OJIP curve represents a plot of fluorescence data on a logarithmic time scale for each sampling day under chilling compared with the control group (day 0). This curve revealed distinct differences dependent on the duration of the chilling treatment. Chilling caused plants to show higher fluorescence intensity at the J-step (2 ms) (V_J_) and lower values at the I-step (V_I_), bringing about a thoroughly changed OJIP curve shape compared to controls (**Figures 3A, B**). Already after 1 day V_J_ showed a distinct higher positive amplitude compared to control plants. After 1 week this parameter doubled (day 8) and remained relatively stable during the progression of the treatment. V_I_ exhibited the highest, but negative, amplitude after 1 day of chilling and seemed to restore to original values during the rest of the treatment. Chilling stressed plants also exhibited apparent positive L bands with a more pronounced amplitude during the progression of the chilling treatment (**Figure 3C**). In contrast, negative K bands with a relative distinct amplitude were already visible after 1 day of treatment and did not change during the progression of the experiment (**Figure 3D**). Finally, chilling also affected difference kinetics ΔW_OI_ showing a clear positive amplitude after 1 day which remained throughout the experiment (**Figure 3E**).

### Chlorophyll and carotenoid content

**Figure 4:**
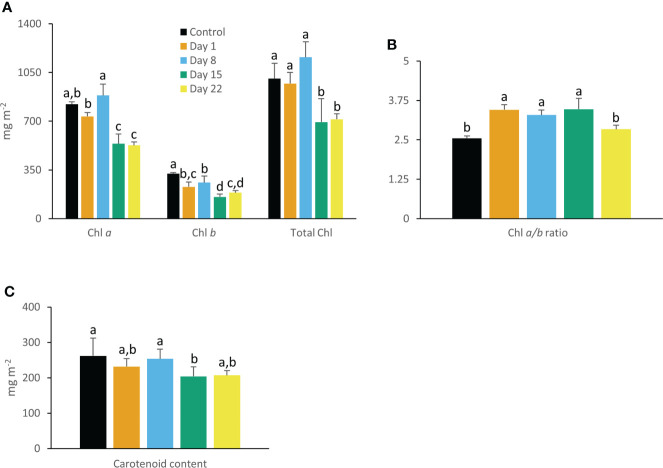
Content (mg m^-2^) of chlorophyll *a*, chlorophyll *b*, total chlorophyll **(A)**, chlorophyll *a/b* ratio **(B)**, and carotenoids **(C)** measured in young fully expanded leaves of *Phalaenopsis* ‘Edessa’ at 0 (control), 1, 8, 15 and 22 days after exposure to chilling conditions (10°C). Data are means ± SE (n = 5 plants). Values were compared among the different time points according to Tukey’s HSD test at p < 0.05 marked by different letters.

After 15 days of growth at 10°C, significant (P < 0.05) lower values in contents of chlorophyll *a*, chlorophyll *b* and total chlorophyll were noticed (**Figure 4A**). The Chl *a/b* ratio initially increased during the first 15 days (P < 0.05) but restored to original values at the end of the experiment (P > 0.05) (**Figure 4B**). Carotenoid content remained relatively stable during progression of the chilling treatment (**Figure 4C**).

### Rubisco activity

**Figure 5:**
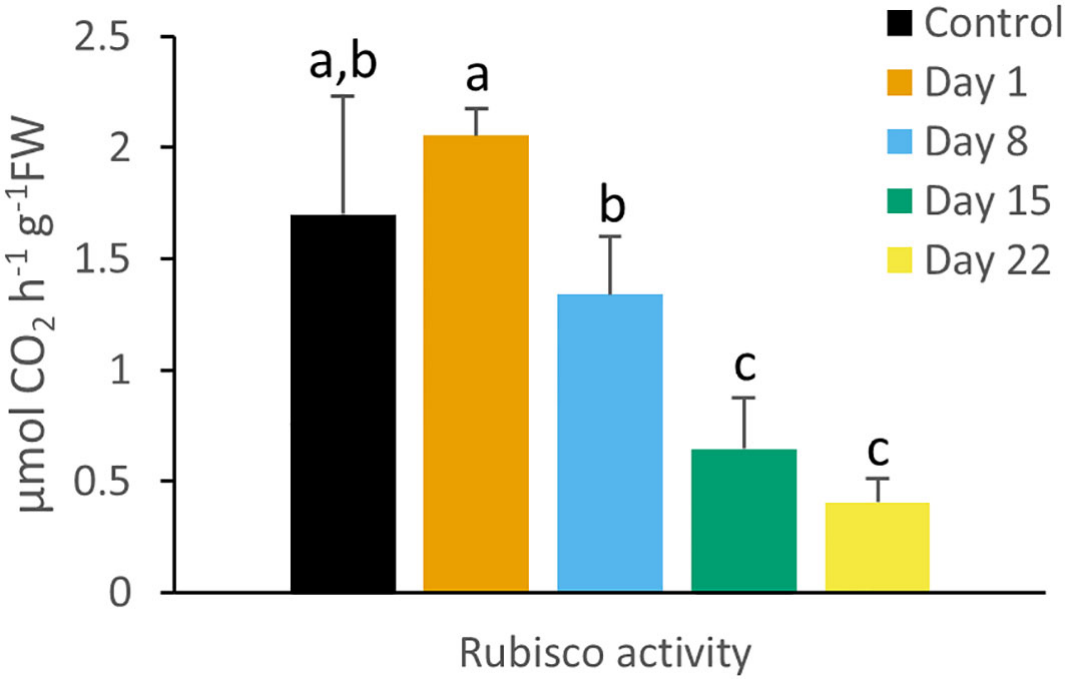
Rubisco enzyme activity (µmol CO_2_ h^-1^ g^-1^FW) measured at 08.00 h in young fully expanded leaves of *Phalaenopsis* ‘Edessa’ at 0 (control), 1, 8, 15 and 22 days after exposure to chilling conditions (10°C). Data are means ± SE (n = 5 plants). Values were compared among the different time points according to Tukey’s HSD test at p < 0.05 marked by different letters.

Rubisco activity showed a declining trend during the cold treatment but only after 2 weeks, activity was significantly (P < 0.05) affected (**Figure 5**).

### Gas exchange measurements

**Figure 6:**
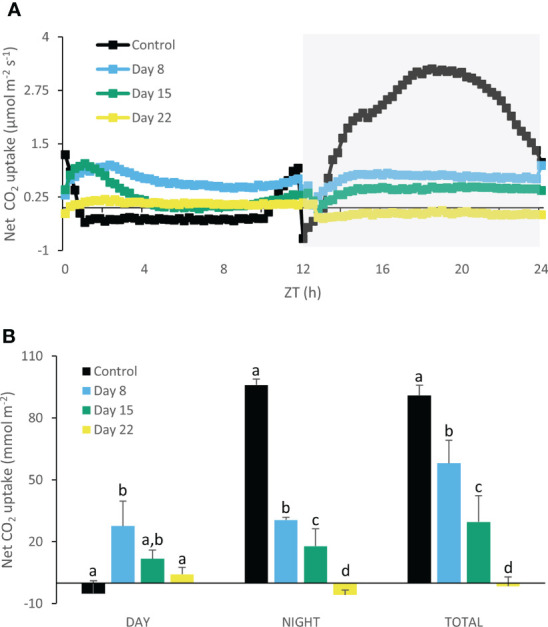
Net 24h CO_2_ uptake (µmol m^-2^ s^-1^) **(A)** for young fully expanded leaves of *Phalaenopsis* ‘Edessa’ at 0 (control), 8, 15 and 22 days after exposure to chilling conditions (10°C). The dark period is indicated in gray. Gas exchange curves are representative of three replicate runs with SE < 15%. Net CO_2_ uptake (mmol m^-2^) **(B)** during the day and night as well as total uptake over the diel cycle derived by integration. Data are means ± SE (n = 3 plants). Values were compared among the different time points according to Tukey’s HSD test at p < 0.05 marked by different letters.

**Figure 7:**
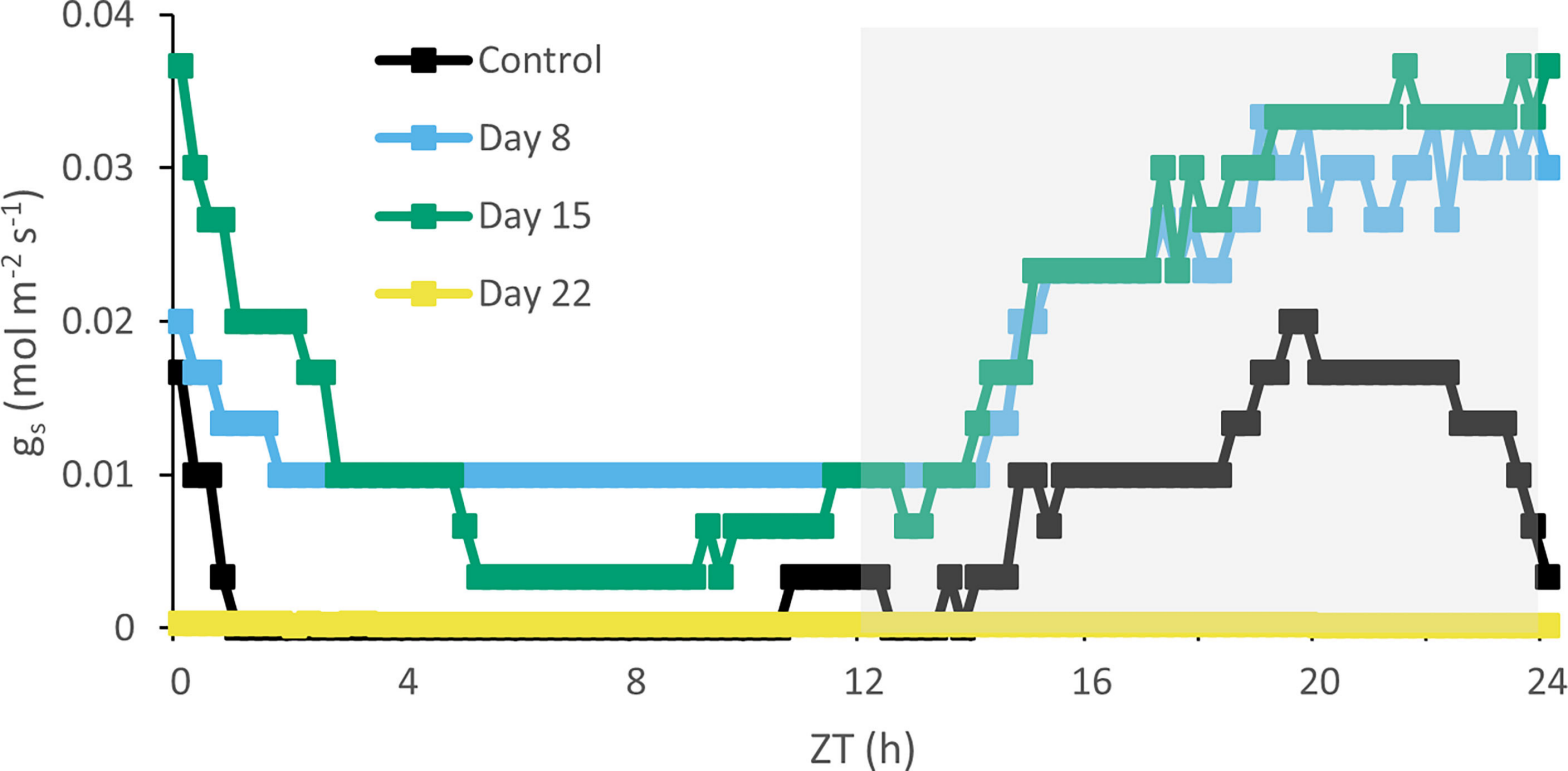
Diel stomatal conductance (mol m^-2^ s^-1^) curves for young fully expanded leaves of *Phalaenopsis* ‘Edessa’ at 0 (control), 8, 15 and 22 days after exposure to chilling conditions (10°C). The dark period is indicated in gray. Curves are representative of three replicate runs with SE < 15%.

A traditional diel CAM pattern was observed in control plants (**Figure 6A**) with carbon fixation during the night (Phase I) and net diurnal CO_2_ loss (Phase III), flanked by two intermediate phases with net CO_2_ uptake during daytime (Phases II and IV). After 8 days of chilling nocturnal uptake was drastically reduced, but a concomitant strong increase in daytime uptake assured that still 65% of total carbon uptake occurred compared to the initial situation (**Figure 6B**). These findings were corroborated by stomatal conductance data showing a consistently higher stomatal conductance during daytime in cold stressed plants (days 8 and 15) (**Figure 7**). In contrast, higher nighttime stomatal conductance did not bring about any carbon gain. A further decline in total carbon uptake (day and night) was noticed after 15 days of chilling, but now only 33% of total carbon uptake occurred compared to the initial situation. After 22 days under cold conditions plants did not show any signs of net assimilation anymore.

**Figure 8:**
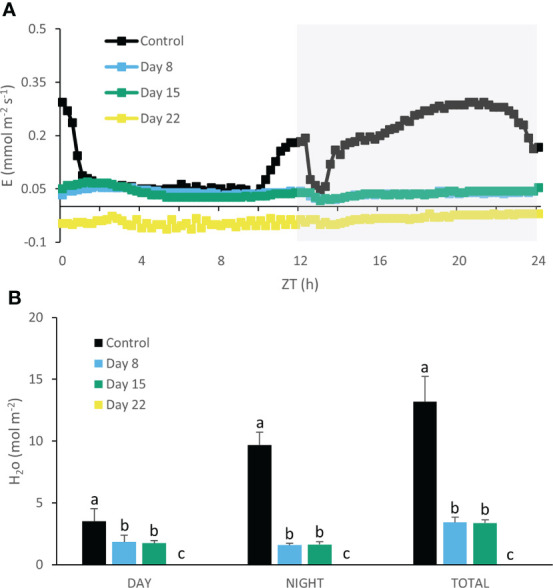
Net 24h transpiration (mmol m^-2^ s^-1^) **(A)** for young fully expanded leaves of *Phalaenopsis* ‘Edessa’ at 0 (control), 8, 15 and 22 days after exposure to chilling conditions (10°C). The dark period is indicated in gray. Gas exchange curves are representative of three replicate runs with SE < 15%. Net H_2_O loss (mol m^-2^) **(B)** during the day and night as well as total loss over the diel cycle derived by integration. Data are means ± SE (n = 3 plants). Values were compared among the different time points according to Tukey’s HSD test at p < 0.05 marked by different letters.

As expected, transpirational water losses matched the CAM CO_2_ uptake pattern in control plants, showing higher rates of transpiration in Phases I, II and IV compared to Phase III (**Figure 8A**). Eight days of chilling caused a drastic reduction in transpiration rate of ca. 400% which remained rather stable during the complete 24h cycle. Seven extra days of chilling (day 15) did not seem to alter transpiration kinetics but after 22 days no net transpiration rate was recorded anymore (**Figure 8B**).

### Metabolites

**Figure 9:**
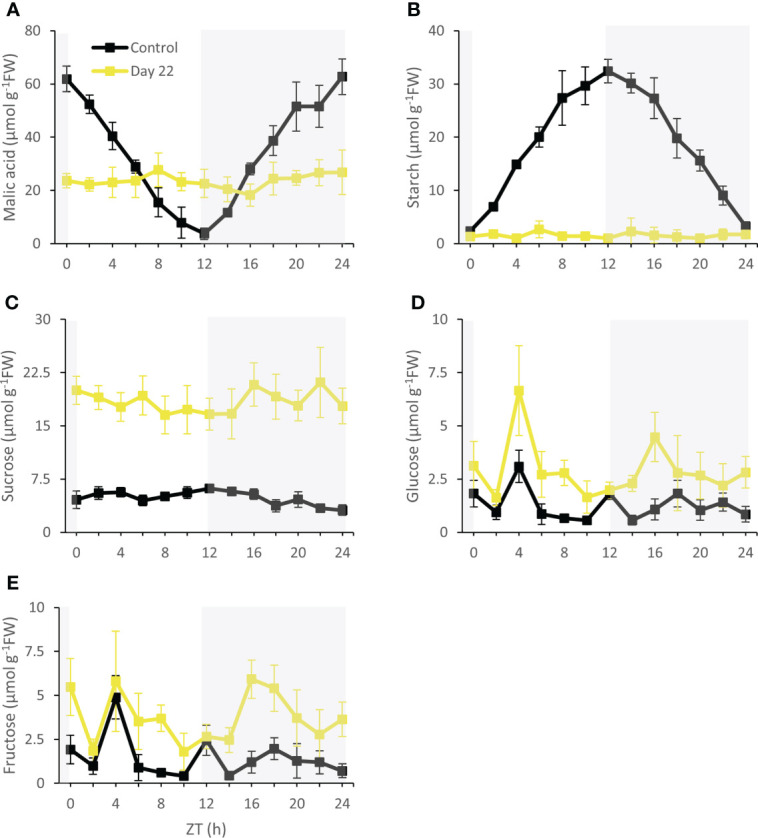
Diel patterns of malic acid (µmol g^-1^FW) **(A)**, starch (µmol g^-1^FW) **(B)**, sucrose (µmol g^-1^FW) **(C)**, glucose (µmol g^-1^FW) **(D)** and fructose (µmol g^-1^FW) **(E)** for young fully expanded leaves of *Phalaenopsis* ‘Edessa’ at 0 (control) and 22 days after exposure to chilling conditions (10°C). The dark period is indicated in gray. Data are means ± SE (n = 5 plants).

Under control conditions 58 ± 3 µmol g^-1^FW malic acid was degraded during the day (ZT0 – ZT12) and 59 ± 4 µmol g^-1^FW was synthesized during the night (ZT12 – ZT24) showing a typical day/night pattern (**Figure 9A**). As expected, starch showed an inverse diel rhythm compared with malic acid and accumulated 30 ± 3 µmol g^-1^FW during the day followed by nocturnal degradation of 29 ± 2 µmol g^-1^FW (**Figure 9B**). After 22 days of chilling malic acid and starch contents showed a rather stable pattern over the complete diel cycle with relatively low mean values of 24 ± 5 µmol g^-1^FW and 2 ± 1 µmol g^-1^FW respectively (**Figures 9A, B**). In contrary, leaf sucrose contents were significantly (P < 0.05) higher after 22 days (18 ± 3 µmol g^-1^FW) compared to controls (5 ± 1 µmol g^-1^FW) (**Figure 9C**). Leaf hexoses i.e. glucose and fructose fluctuated strongly over the diel phase and also seemed to be pushed up by the cold treatment by a factor of ca. 2.5 (P < 0.05) (**Figures 9D, E**).

## Discussion

### Short term chilling effects on the efficiency of the light reactions

Already after 24 h chilling clearly affected the light reactions in the leaves of *Phalaenopsis* ‘Edessa’. The significant increase in reduction flux (Re_0_/RC) after 1 day of chilling possibly highlights overreduction on the electron acceptor side. The rapid inactivation of some PSII RCs, becoming non-Q_A_-reducing centers, can explain the observed increase in Tr_0_/RC after one day of chilling. Our results also indicated that the performance index for absorption (PI_abs_) was a sensitive parameter to indicate chilling stress by showing already a tough decline after one day of cold treatment. Photo-inhibitory effects were not yet present since the maximum quantum efficiency of PSII photochemistry (F_v_/F_m_) was not significantly affected in the short term (**Figures 1A, B**). The massive reduction of PI_abs_, which serves as a sensitive indicator of plant vitality (Živčák et al., 2008), is generally related with an overall downregulation of PSII related energy fluxes as a physiological response to chilling stress (Liang et al., 2019). However, our study shows that the picture is more complicated on the short term as several fluxes were significantly upregulated (i.e. Tr_0_/RC and Re_0_/RC). Total chlorophyll, chlorophyll *a*, and carotenoid content remained unaffected whilst chlorophyll *b* levels dropped significantly after 1 day of chilling which caused the ratio between chl *a* and chl *b* to increase (**Figures 4A-C**).

Chilling treated plants showed the minimum fluorescence intensity at the I-phase (V_I_, 30 ms) (**Figures 3A, B**) already after day 1, indicating that chilling induced a higher probability by which electrons move from PSII to PSI acceptor side (Faseela et al., 2020). However, an increase in ΔW_OI_ was also observed which indicates that electron transport from PSII towards plastoquinone (PQ) (i.e. between Q_A_ and Q_B_) was substantially blocked (Yusuf et al., 2010) (**Figure 3E**). The K-band (300 µs, ΔW_OJ_) is closely related with the imbalance of electron flow from donor side to acceptor side in PSII RCs (Srivastava et al., 1997). Different studies revealed that negative K-band values act as an indication of better performance of plants under stressful environmental conditions (Yusuf et al., 2010; Krüger et al., 2014; Żurek et al., 2014; Begović et al., 2016). Moreover, Venkatesh et al. (2012) and Kalaji et al. (2016) reported that a negative K-band indicates an intactness of the oxygen-evolving complex (OEC) and thus, functional PSII antenna size. Also in our study OEC probably contributed more to the donation of electrons towards PSII than in control plants (**Figure 3D**) what is considered to be a competent mechanism to improve tolerance under short-term chilling (Mlinarić et al., 2021).

### Mid term effects on the efficiency of the light reactions

Two possible explanations can be presented for the observed increase in ABS/RC under chilling stress on the longer term (3 weeks) (**Figure 2A**): (1) inactivation of some PSII RCs, considering that the ABS/RC ratio is calculated as the total number of photons absorbed by Chl molecules in all RCs divided by the total number of active RCs, or (2) an increase in antenna size (Liang et al., 2007; Gupta et al., 2021). The latter seems to be less plausible since total chlorophyll content was not affected in the first week (days 1 and 8) and significant lower values were obtained after 15 days of chilling (**Figure 4A**). The decline in total chlorophyll content was mainly due to a significant decrease in Chl *a* only after 15 days of treatment, which can also reflect a decline in active RCs. The inactivation of RCs due to chilling stress is additionally evidenced by the observed decline in F_v_/F_m_. Decreased values of F_v_/F_m_ below 0.75 indicate that PSII has been damaged (Krause and Weis, 1991) which was observed after 1 week (day 8) of chilling treatment. The significant increase in Di_0_/RC after 15 days and decrease in Et_0_/RC indicate a reduction in efficiency of photosynthesis by showing that the excess excitation energy was mostly dissipated (Strasser et al., 2004; Yusuf et al., 2010). Down-regulation of photochemical activity together with a significant increase in thermal dissipation may play a critical role in protecting plants exposed to chilling stress from over-excitation and photodamage, a process called photoinhibition (Gilmore, 1997; Hu et al., 2008; Zhang et al., 2010).

After 8 days of chilling plants also exhibited a remarkable higher fluorescence intensity at the J-phase (V_J_, 2 ms) (**Figures 3A, B**) which usually reflects the accumulation of ${\text{QA}}_{-}^{\;}$ (Strasser et al., 1995; Lazár and Ilík, 1997), possibly due to a decrease in electron transport beyond ${\text{QA}}_{-}^{\;}$ (Haldimann and Strasser, 1999). This observation also coincidences with the observed increase in ΔW_OI_ which indicates that electron transport from PSII towards plastoquinone (PQ) (i.e. between Q_A_ and Q_B_) was substantially blocked (Yusuf et al., 2010) (**Figure 3E**). These results also align with the massive increase in the energy trapped and the significant decline in electron transport flux. These possible obstructions in the electron transport chain will also impact proton homeostasis around the thylakoid membrane. Provision of sufficient protons into the thylakoid lumen is key to drive ATP synthase (ATPase) and can occur in two different ways: (1) *via* the OEC which converts water into oxygen, protons and electrons or (2) *via* the electron transport flux (Et_0_/RC) which causes two protons being pumped into the thylakoid lumen per electron in the cytochrome b_6_f complex. It has been reported that low temperatures can cause thylakoids to decouple in chilling-sensitive plants (Peeler and Naylor, 1988; Terashima et al., 1989; Terashima et al., 1991a; Terashima et al., 1991b). Low temperatures can directly rigidify the thylakoid membrane, thereby affecting the activity of the photosynthetic system already in the short term. It could be expected that the fluidity of thylakoid membranes in this tropical CAM plant was drastically altered under chilling conditions, hence decreasing the efficiency of photosynthesis (Liu et al., 2018; Mazur et al., 2020). However, photoprotection has been noticed in chilled tobacco plants by establishment of an even higher proton gradient (ΔpH) across the thylakoid membrane by reduced activity of chloroplastic ATPase (Yang et al., 2018). Oxidation of PQH_2_ at the cytochrome b_6_f complex decelerated due to the higher ΔpH, which limited the electron transfer to PSI avoiding overexcitation (Suorsa et al., 2012; Tikkanen and Aro, 2014; Tikkanen et al., 2015). A second approach to maintain ΔpH, hence activating photoprotection processes, in limited environmental conditions is the switch between linear and cyclic electron transport. Both linear and cyclic electron transport around PSI are driven by the light reactions. Cyclic electron flow contributes to the development of a ΔpH, without any accumulation of NADPH in chloroplasts, whereas PSI linear electron flow produces both ATP and NADPH (Shikanai, 2007). Evidence was presented by Clarke and Johnson (2001) that cyclic electron transport around PSI acts to maintain membrane energization at low temperatures (10°C) in leaves of barley (*Hordeum vulgare*). Cyclic electron flow might be favoured because of a protective role owing to increased thylakoid acidification which also initiates non-photochemical dissipation of excess excitation energy (Horton et al., 1996; Johnson, 2005). This also aligns with our observed increase in dissipation flux (Di_0_/RC) after 1 week (day 8) of chilling.

Throughout the following weeks (days 8, 15, and 22) I-band (V_I_) variable fluorescence was restored to values similar to control plants (i.e. probability by which electrons move from PSII to PSI acceptor side) which is consistent with the observed data of electron transport and reduction flux. The L-band (150 µs, ΔW_OK_) can be considered to depict energetic connectivity of the antennae to PSII units (Yusuf et al., 2010; Mlinarić et al., 2017), indicating excitation energy utilization and system stability of PSII units (Strasser et al., 2004). The existence of a positive L-band in the chilling treated plants (**Figure 3C**) pointed to an inferior performance of antennae connectivity, bringing about disturbed energy transfer compared to control plants after 1 week (Srivastava et al., 1997; Liang et al., 2019). Therefore, PSII units of chilling treated plants had lower stability and became even more fragile during the progression of the experiment according to the change in amplitude of the L-band. These data align with the strong decrease in the maximum quantum efficiency of PSII photochemistry (F_v_/F_m_).

### Chilling effects on carbon fixation and partitioning

Under chilling conditions CAM plasticity initially assured that still ca. 65% of CO_2_ was captured after 1 week compared to unstressed plants. The massive reduction in nocturnal CO_2_ uptake was partly compensated by a strong increase in daytime uptake (**Figures 6A, B**). These results are in line with an unaffected Rubisco activity after 8 days of chilling. Phosphoenolpyruvate carboxylase (PEPC) activity is likely to suffer more from chilling temperatures than Rubisco activity as indicated by specific temperature response curves of the C_4_ species *Paspalum dilatatum* at 10°C (ca. 50 nmol CO_2_ h^-1^ g^-1^FW and ca. 400 nmol CO_2_ h^-1^ g^-1^FW respectively) (Cavaco et al., 2003). This view is also consistent with the observed higher degree of daytime stomatal opening allowing more Rubisco uptake whilst increased stomatal conductance did not result in any nocturnal carbon gain (**Figure 7**). After two weeks of cold treatment stomatal conductance was still high but reduced Rubisco activity led to a significant decline in CO_2_ uptake. Reduced Rubisco activity also entails a diminished consumption of ATP and NADPH by the Calvin-Benson cycle, possibly providing negative feedback on PSII operating efficiency and increasing the extent of PSII photoinhibition. Limited CO_2_ fixation causes NADP^+^ levels to drop, which in turn stimulate the generation of ROS that suppress protein synthesis for the repair of PSII in chloroplasts (Allakhverdiev and Murata, 2004; Takahashi and Murata, 2006; Murata et al., 2007; Takahashi and Murata, 2008).

Three weeks of chilling caused stomatal conductance and CO_2_ uptake to diminish to near zero levels, in accordance with an invariable leaf malic acid content (**Figure 9A**), indicating that plants were not successful in cold acclimation on the longer term. It has been reported that the affinity of malic enzyme (ME) for malate is lower due to chilling conditions (10 to 15°C). The conversion of malate to pyruvate will be scanty and this might favor malate accumulation (Brandon, 1967; Brandon and van Boekel-Mol, 1973). In addition, tonoplast permeability is also affected under low temperatures which can block both vacuolar malate influx or efflux (Kliemchen et al., 1993). In addition, leaf starch content was drastically reduced to near zero levels, whilst sucrose was dramatically pushed up by a factor of ca. 4 (**Figures 9B, C**). A similar increase was noticed for both glucose and fructose (**Figures 9D, E**). This reduced starch: soluble sugars ratio suggests that photoassimilation under chilling was deficient for maintaining growth since stock components were remobilized and not restocked (Oliveira and Peñuelas, 2005). Maintaining sucrose pools rather than starch might partially be explained by considerations of osmoprotection. Many plants regulate their osmotic potential through accumulation of solutes inside their cells, such as sucrose, glucose and fructose (Couée et al., 2006; Ruelland et al., 2009; Buchanan et al., 2015). Carvalho et al. (2013) observed a higher leaf glucose content *in vitro* in the bromeliad *Nidularium minutum* at growth under 10°C for six months compared to plants grown at 25°C. Moreover, an increase in activity of enzymes involved in sucrose synthesis [i.e. sucrose-phosphate synthase (SPS)] was observed in *A. thaliana* and cabbage (*Brassica oleracea*) after treatment at 4°C for 24 hours and 5°C for 72 hours respectively (Sasaki et al., 2001; Nägele et al., 2011). Higher activity of SPS indicates a key role in sugar accumulation and acquisition of cold tolerance (Guy et al., 1992; Nievola et al., 2017). Furthermore, when comparing enzyme biochemistry and requisite transport steps that are potentially involved in the synthesis and degradation of soluble sugars compared to starch, Black et al. (1996) postulated a lower energy cost in terms of ATP for CAM species that use soluble sugars as the source of PEP. Especially under particular abiotic stress conditions of drought and low light Ceusters et al. (2009; 2011) already demonstrated an increase in leaf sucrose content with a concomitant decrease in starch in the CAM bromeliad *Aechmea* ‘Maya’.

## Conclusion

Chilling (10°C) already affected the light reactions in the CAM plant *Phalaenopsis* ‘Edessa’ after 24h. Whilst the potential efficiency of photosystem II (PSII) (F_v_/F_m_) was not yet influenced, a massive decrease in the performance index (PI_abs_) was noticed. This decrease did not depict an overall downregulation of PSII related energy fluxes since energy absorption and dissipation remained uninfluenced whilst the trapped energy and reduction flux were upregulated. This might point to the presence of short-term adaptation mechanisms to chilling stress. However, in the longer term the electron transport chain from PSII to PSI was affected, impacting both ATP and NADPH provision. To avoid over-excitation and photodamage plants showed a massive increase in thermal dissipation. Limited CO_2_ fixation by Rubisco after two weeks of chilling initiated a negative feedback mechanism on PSII activity which led to the occurrence of severe PSII photoinhibition. These considerations are also in line with the carbon fixation data showing initial signs of cold adaptation by achieving comparable Rubisco activity levels compared to unstressed plants but increasing daytime stomatal opening in order to capture a higher proportion of CO_2_ during daytime. However, in accordance with the light reactions data, Rubisco activity declined and stomatal conductance and CO_2_ uptake diminished to near zero levels after three weeks, indicating that plants were not successful in cold acclimation on the longer term.

## Data availability statement

The raw data supporting the conclusions of this article will be made available by the authors, without undue reservation.

## Author contributions

NC, RV and JC proposed the conceptual framework for the study. NC and SD performed the experimental analyses. Data collection and analysis were performed by SD, NC, RV and JC. Data interpretation and writing the manuscript were performed by SD, RV and JC. All authors contributed to the article and approved the submitted version.

## Funding

This research was supported by the Research Fund KU Leuven.

## Acknowledgments

Microflor NV is acknowledged for supplying plant material.

## Conflict of interest

The authors declare that the research was conducted in the absence of any commercial or financial relationships that could be construed as a potential conflict of interest.

## Publisher’s note

All claims expressed in this article are solely those of the authors and do not necessarily represent those of their affiliated organizations, or those of the publisher, the editors and the reviewers. Any product that may be evaluated in this article, or claim that may be made by its manufacturer, is not guaranteed or endorsed by the publisher.

## References

[B1] Agati G. Mazzinghi P. Lipucci di Paola M. Fusi F. Cecchi G. (1996). The F685/F730 chlorophyll fluorescence ratio as indicator of chilling stress in plants. J. Plant Physiol. 148, 384–390. doi: 10.1016/S0176-1617(96)80270-7

[B2] Allakhverdiev S. I. Murata N. (2004). Environmental stress inhibits the synthesis *de novo* of proteins involved in the photodamage-repair cycle of photosystem II in synechocystis sp. PCC 6803. Biochim. Biophys. Acta 1657, 23–32. doi: 10.1016/j.bbabio.2004.03.003 15238209

[B3] Allen D. J. Ort D. R. (2001). Impacts of chilling temperatures on photosynthesis in warm-climate plants. Trends Plant Sci. 6, 36–42. doi: 10.1016/S1360-1385(00)01808-2 11164376

[B4] Arnell N. W. Lowe J. A. Challinor A. J. Osborn T. J. (2019). Global and regional impacts of climate change at different levels of global temperature increase. Climatic Change 155, 377–391. doi: 10.1007/s10584-019-02464-z

[B5] Begović L. Mlinarić S. Antunović Dunić J. Katanić Z. Lončarić Z. Lepeduš H.. (2016). Response of lemna minor l. @ to short-term cobalt exposure: The effect on photosynthetic electron transport chain and induction of oxidative damage. Aquat. Toxicol. 175, 117–126. doi: 10.1016/j.aquatox.2016.03.009 27015565

[B6] Bhandari S. R. Kim Y. H. Lee J. G. (2018). Detection of temperature stress using chlorophyll fluorescence parameters and stress-related chlorophyll and proline content in paprika (Capsicum annuum l.) seedlings. Hortic. Sci. Technol. 36 (5), 619–629. doi: 10.12972/kjhst.20180062

[B7] Black C. C. Chen J.-Q. Doong R. L. Angelov M. N. Sung S. J. S. (1996). “Alternative carbohydrate reserves used in the daily cycle of crassulacean acid metabolism,” in Crassulacean acid metabolism: Biochemistry, ecophysiology and evolution ecological studies. Eds. Winter K. Smith J. A. C. (Berlin, Heidelberg: Springer), 31–45. doi: 10.1007/978-3-642-79060-7_3

[B8] Borland A. M. Barrera Zambrano V. A. Ceusters J. Shorrock K. (2011). The photosynthetic plasticity of crassulacean acid metabolism: an evolutionary innovation for sustainable productivity in a changing world. New Phytol. 191, 619–633. doi: 10.1111/j.1469-8137.2011.03781.x 21679188

[B9] Borland A. M. Técsi L. I. Leegood R. C. Walker R. P. (1998). Inducibility of crassulacean acid metabolism (CAM) in clusia species; physiological/biochemical characterisation and intercellular localization of carboxylation and decarboxylation processes in three species which exhibit different degrees of CAM. Planta 205, 342–351. doi: 10.1007/s004250050329

[B10] Brandon P. C. (1967). Temperature features of enzymes affecting crassulacean acid metabolism. Plant Physiol. 42, 977–984. doi: 10.1104/pp.42.7.977 16656606 PMC1086659

[B11] Brandon P. C. van Boekel-Mol T. N. (1973). Properties of purified malic enzyme in relation to crassulacean acid metabolism. Eur. J. Biochem. 35, 62–69. doi: 10.1111/j.1432-1033.1973.tb02810.x 4145921

[B12] Buchanan B. B. Gruissem W. Jones R. L. (2015). Biochemistry and molecular biology of plants (The Atrium, Southern Gate, Chichester, West Sussex, P019 8SQ, UK: John Wiley & Sons).

[B13] Byrd G. T. Ort D. R. Ogren W. L. (1995). The effects of chilling in the light on ribulose-1,5-Bisphosphate Carboxylase/Oxygenase activation in tomato (Lycopersicon esculentum mill.). Plant Physiol. 107, 585–591. doi: 10.1104/pp.107.2.585 12228384 PMC157162

[B14] Carvalho C. P. Hayashi A. H. Braga M. R. Nievola C. C. (2013). Biochemical and anatomical responses related to the in vitro survival of the tropical bromeliad nidularium minutum to low temperatures. Plant Physiol. Biochem. 71, 144–154. doi: 10.1016/j.plaphy.2013.07.005 23917072

[B15] Cavaco A. M. Da Silva A. B. Arrabaça M. C. (2003). Effects of long-term chilling on growth and photosynthesis of the C4 gramineae paspalum dilatatum. Physiologia Plantarum 119, 87–96. doi: 10.1034/j.1399-3054.2003.00148.x

[B16] Cen Y.-P. Sage R. F. (2005). The regulation of rubisco activity in response to variation in temperature and atmospheric CO2 partial pressure in sweet potato. Plant Physiol. 139, 979–990. doi: 10.1104/pp.105.066233 16183840 PMC1256011

[B17] Ceusters J. Borland A. M. Godts C. Londers E. Croonenborghs S. Van Goethem D.. (2011). Crassulacean acid metabolism under severe light limitation: a matter of plasticity in the shadows? J. Exp. Bot. 62, 283–291. doi: 10.1093/jxb/erq264 20861137

[B18] Ceusters J. Borland A. M. Londers E. Verdoodt V. Godts C. De Proft M. P. (2008). Diel shifts in carboxylation pathway and metabolite dynamics in the CAM bromeliad aechmea ‘Maya’ in response to elevated CO2. Ann. Bot. 102, 389–397. doi: 10.1093/aob/mcn105 18593689 PMC2701804

[B19] Ceusters J. Borland A. M. Londers E. Verdoodt V. Godts C. De Proft M. P. (2009). Differential usage of storage carbohydrates in the CAM bromeliad aechmea ‘Maya’ during acclimation to drought and recovery from dehydration. Physiologia Plantarum 135, 174–184. doi: 10.1111/j.1399-3054.2008.01186.x 19077141

[B20] Ceusters N. Ceusters J. Hurtado-Castano N. Dever L. V. Boxall S. F. Kneřová J.. (2021). Phosphorolytic degradation of leaf starch *via* plastidic α-glucan phosphorylase leads to optimized plant growth and water use efficiency over the diel phases of crassulacean acid metabolism. J. Exp. Bot. 72, 4419–4434. doi: 10.1093/jxb/erab132 33754643 PMC8266541

[B21] Ceusters N. Valcke R. Frans M. Claes J. E. Van den Ende W. Ceusters J. (2019). Performance index and PSII connectivity under drought and contrasting light regimes in the CAM orchid phalaenopsis. Front. Plant Sci. 10. doi: 10.3389/fpls.2019.01012 PMC6691161 31447875

[B22] Chen T. H. H. (1994). Plant adaptation to low temperature stress. Can. J. Plant Pathol. 16, 231–236. doi: 10.1080/07060669409500760

[B23] Chen L. Lin Q. Nose A. (2002). A comparative study on diurnal changes in metabolite levels in the leaves of three crassulacean acid metabolism (CAM) species, ananas comosus, kalanchoë daigremontiana and k. pinnata. J. Exp. Bot. 53, 341–350. doi: 10.1093/jexbot/53.367.341 11807138

[B24] Chiang F. Mazdiyasni O. AghaKouchak A. (2021). Evidence of anthropogenic impacts on global drought frequency, duration, and intensity. Nat. Commun. 12, 2754. doi: 10.1038/s41467-021-22314-w 33980822 PMC8115225

[B25] Chondrogiannis C. Grammatikopoulos G. (2016). Photosynthesis in developing leaf of juveniles and adults of three Mediterranean species with different growth forms. Photosynth Res. 130, 427–444. doi: 10.1007/s11120-016-0276-4 27220729

[B26] Clarke J. E. Johnson G. N. (2001). *In vivo* temperature dependence of cyclic and pseudocyclic electron transport in barley. Planta 212, 808–816. doi: 10.1007/s004250000432 11346955

[B27] Couée I. Sulmon C. Gouesbet G. El Amrani A. (2006). Involvement of soluble sugars in reactive oxygen species balance and responses to oxidative stress in plants. J. Exp. Bot. 57, 449–459. doi: 10.1093/jxb/erj027 16397003

[B28] DePaoli H. C. Borland A. M. Tuskan G. A. Cushman J. C. Yang X. (2014). Synthetic biology as it relates to CAM photosynthesis: challenges and opportunities. J. Exp. Bot. 65, 3381–3393. doi: 10.1093/jxb/eru038 24567493

[B29] Dong S. Beckles D. M. (2019). Dynamic changes in the starch-sugar interconversion within plant source and sink tissues promote a better abiotic stress response. J. Plant Physiol. 234–235, 80–93. doi: 10.1016/j.jplph.2019.01.007 30685652

[B30] ElSayed A. I. Rafudeen M. S. Golldack D. (2014). Physiological aspects of raffinose family oligosaccharides in plants: protection against abiotic stress. Plant Biol. 16, 1–8. doi: 10.1111/plb.12053 23937337

[B31] Farrar J. F. (1988). Temperature and the partitioning and translocation of carbon. Symp Soc. Exp. Biol. 42, 203–235. 3077858

[B32] Faseela P. Sinisha A. K. Brestič M. Puthur J. T. (2020). Chlorophyll a fluorescence parameters as indicators of a particular abiotic stress in rice. Photosynt. 58, 293–300. doi: 10.32615/ps.2019.147

[B33] Fowler S. Thomashow M. F. (2002). Arabidopsis transcriptome profiling indicates that multiple regulatory pathways are activated during cold acclimation in addition to the CBF cold response Pathway[W]. Plant Cell 14, 1675–1690. doi: 10.1105/tpc.003483 12172015 PMC151458

[B34] Gilmore A. M. (1997). Mechanistic aspects of xanthophyll cycle-dependent photoprotection in higher plant chloroplasts and leaves. Physiologia Plantarum 99, 197–209. doi: 10.1111/j.1399-3054.1997.tb03449.x

[B35] Greer D. H. Berry J. A. Björkman O. (1986). Photoinhibition of photosynthesis in intact bean leaves: role of light and temperature, and requirement for chloroplast-protein synthesis during recovery. Planta 168, 253–260. doi: 10.1007/BF00402971 24232029

[B36] Gupta R. Sharma R. D. Rao Y. R. Siddiqui Z. H. Verma A. Ansari M. W.. (2021). Acclimation potential of noni (Morinda citrifolia l.) plant to temperature stress is mediated through photosynthetic electron transport rate. Plant Signaling Behav. 16, 1865687. doi: 10.1080/15592324.2020.1865687 PMC7889107 33356839

[B37] Guy C. L. Huber J. L. A. Huber S. C. (1992). Sucrose phosphate synthase and sucrose accumulation at low temperature 1. Plant Physiol. 100, 502–508. doi: 10.1104/pp.100.1.502 16652990 PMC1075578

[B38] Habibi G. (2019). Effects of high light and chilling stress on photosystem II efficiency of aloe vera l. plants probing by chlorophyll a fluorescence measurements. Iran J. Sci. Technol. Trans. Sci. 43, 7–13. doi: 10.1007/s40995-017-0378-7

[B39] Haldimann P. (1999). How do changes in temperature during growth affect leaf pigment composition and photosynthesis in zea mays genotypes differing in sensitivity to low temperature? J. Exp. Bot. 50, 543–550. doi: 10.1093/jxb/50.333.543

[B40] Haldimann P. Strasser R. J. (1999). Effects of anaerobiosis as probed by the polyphasic chlorophyll a fluorescence rise kinetic in pea (Pisum sativum l.). Photosynthesis Res. 62, 67–83. doi: 10.1023/A:1006321126009

[B41] Horton P. Ruban A. V. Walters R. G. (1996). REGULATION OF LIGHT HARVESTING IN GREEN PLANTS. Annu. Rev. Plant Physiol. Plant Mol. Biol. 47, 655–684. doi: 10.1146/annurev.arplant.47.1.655 15012304

[B42] Hou W. Sun A. H. Chen H. L. Yang F. S. Pan J. L. Guan M. Y. (2016). Effects of chilling and high temperatures on photosynthesis and chlorophyll fluorescence in leaves of watermelon seedlings. Biol. Plant 60, 148–154. doi: 10.1007/s10535-015-0575-1

[B43] Hurry V. M. Malmberg G. Gardestrom P. Oquist G. (1994). Effects of a short-term shift to low temperature and of long-term cold hardening on photosynthesis and ribulose-1,5-Bisphosphate Carboxylase/Oxygenase and sucrose phosphate synthase activity in leaves of winter rye (Secale cereale l.). Plant Physiol. 106, 983–990. doi: 10.1104/pp.106.3.983 12232378 PMC159622

[B44] Hurry V. M. Strand A. Tobiaeson M. Gardestrom P. Oquist G. (1995). Cold hardening of spring and winter wheat and rape results in differential effects on growth, carbon metabolism, and carbohydrate content. Plant Physiol. 109, 697–706. doi: 10.1104/pp.109.2.697 12228623 PMC157638

[B45] Hu W. H. Song X. S. Shi K. Xia X. J. Zhou Y. H. Yu J. Q. (2008). Changes in electron transport, superoxide dismutase and ascorbate peroxidase isoenzymes in chloroplasts and mitochondria of cucumber leaves as influenced by chilling. Photosynthetica 46, 581. doi: 10.1007/s11099-008-0098-5

[B46] Hussain H. A. Hussain S. Khaliq A. Ashraf U. Anjum S. A. Men S.. (2018). Chilling and drought stresses in crop plants: Implications, cross talk, and potential management opportunities. Front. Plant Sci. 9. doi: 10.3389/fpls.2018.00393 PMC5902779 29692787

[B47] Johnson G. N. (2005). Cyclic electron transport in C3 plants: fact or artefact? J. Exp. Bot. 56, 407–416. doi: 10.1093/jxb/eri106 15647314

[B48] Johnson N. C. Xie S.-P. Kosaka Y. Li X. (2018). Increasing occurrence of cold and warm extremes during the recent global warming slowdown. Nat. Commun. 9, 1724. doi: 10.1038/s41467-018-04040-y 29712890 PMC5928063

[B49] Jung S.-H. Lee J.-Y. Lee D.-H. (2003). Use of SAGE technology to reveal changes in gene expression in arabidopsis leaves undergoing cold stress. Plant Mol. Biol. 52, 553–567. doi: 10.1023/A:1024866716987 12956526

[B50] Kalaji H. M. Jajoo A. Oukarroum A. Brestic M. Zivcak M. Samborska I. A.. (2016). Chlorophyll a fluorescence as a tool to monitor physiological status of plants under abiotic stress conditions. Acta Physiol. Plant 38, 102. doi: 10.1007/s11738-016-2113-y

[B51] Kingston-Smith A. H. Harbinson J. Williams J. Foyer C. H. (1997). Effect of chilling on carbon assimilation, enzyme activation, and photosynthetic electron transport in the absence of photoinhibition in maize leaves. Plant Physiol. 114, 1039–1046. doi: 10.1104/pp.114.3.1039 12223758 PMC158392

[B52] Kliemchen A. Schomburg M. Galla H.-J. Lüttge U. Kluge M. (1993). Phenotypic changes in the fluidity of the tonoplast membrane of crassulacean-acid-metabolism plants in response to temperature and salinity stress. Planta 189, 403–409. doi: 10.1007/BF00194438 24178498

[B53] Kodra E. Steinhaeuser K. Ganguly A. R. (2011). Persisting cold extremes under 21st-century warming scenarios. Geophysical Res. Lett. 38. doi: 10.1029/2011GL047103

[B54] Krause G. H. Weis E. (1991). Chlorophyll fluorescence and photosynthesis: The basics. Annu. Rev. Plant Physiol. Plant Mol. Biol. 42, 313–349. doi: 10.1146/annurev.pp.42.060191.001525

[B55] Krüger G. H. J. De Villiers M. F. Strauss A. J. de Beer M. van Heerden P. D. R. Maldonado R.. (2014). Inhibition of photosystem II activities in soybean (Glycine max) genotypes differing in chilling sensitivity. South Afr. J. Bot. 95, 85–96. doi: 10.1016/j.sajb.2014.07.010

[B56] Lazár D. Ilík P. (1997). High-temperature induced chlorophyll fluorescence changes in barley leaves comparison of the critical temperatures determined from fluorescence induction and from fluorescence temperature curve. Plant Sci. 124, 159–164. doi: 10.1016/S0168-9452(97)04602-5

[B57] Lemoine R. La Camera S. Atanassova R. Dédaldéchamp F. Allario T. Pourtau N.. (2013). Source-to-sink transport of sugar and regulation by environmental factors. Front. Plant Sci. 4. doi: 10.3389/fpls.2013.00272 PMC3721551 23898339

[B58] Liang Y. Chen H. Tang M.-J. Yang P.-F. Shen S.-H. (2007). Responses of jatropha curcas seedlings to cold stress: photosynthesis-related proteins and chlorophyll fluorescence characteristics. Physiologia Plantarum 131, 508–517. doi: 10.1111/j.1399-3054.2007.00974.x 18251888

[B59] Liang H.-Z. Zhu F. Wang R.-J. Huang X.-H. Chu J.-J. (2019). Photosystem II of ligustrum lucidum in response to different levels of manganese exposure. Sci. Rep. 9, 12568. doi: 10.1038/s41598-019-48735-8 31467311 PMC6715691

[B60] Li Q. Chen L.-S. Jiang H.-X. Tang N. Yang L.-T. Lin Z.-H.. (2010). Effects of manganese-excess on CO2 assimilation, ribulose-1,5-bisphosphate carboxylase/oxygenase, carbohydrates and photosynthetic electron transport of leaves, and antioxidant systems of leaves and roots in citrus grandisseedlings. BMC Plant Biol. 10, 42. doi: 10.1186/1471-2229-10-42 20205939 PMC2848762

[B61] Lim S. D. Lee S. Choi W.-G. Yim W. C. Cushman J. C. (2019). Laying the foundation for crassulacean acid metabolism (CAM) biodesign: Expression of the C4 metabolism cycle genes of CAM in arabidopsis. Front. Plant Sci. 10. doi: 10.3389/fpls.2019.00101 PMC6378705 30804970

[B62] Lin M.-J. Hsu B.-D. (2004). Photosynthetic plasticity of phalaenopsis in response to different light environments. J. Plant Physiol. 161, 1259–1268. doi: 10.1016/j.jplph.2004.05.009 15602817

[B63] Lin K. H. Hwang W. C. Lo H. F. (2007). Chilling stress and chilling tolerance of sweet potato as sensed by chlorophyll fluorescence. Photosynthetica 45, 628–632. doi: 10.1007/s11099-007-0108-z

[B64] Liu X. Zhou Y. Xiao J. Bao F. (2018). Effects of chilling on the structure, function and development of chloroplasts. Front. Plant Sci. 9. doi: 10.3389/fpls.2018.01715 PMC6262076 30524465

[B65] Lundmark M. Cavaco A. M. Trevanion S. Hurry V. (2006). Carbon partitioning and export in transgenic arabidopsis thaliana with altered capacity for sucrose synthesis grown at low temperature: a role for metabolite transporters. Plant Cell Environ. 29, 1703–1714. doi: 10.1111/j.1365-3040.2006.01543.x 16913860

[B66] Mazur R. Gieczewska K. Kowalewska Ł. Kuta A. Proboszcz M. Gruszecki W. I.. (2020). Specific composition of lipid phases allows retaining an optimal thylakoid membrane fluidity in plant response to low-temperature treatment. Front. Plant Sci. 11. doi: 10.3389/fpls.2020.00723 PMC7291772 32582253

[B67] McConnell D. Sheehan T. (1978). Anatomical aspects of chilling injury to leaves of phalaenopsis bl. Am. Soc. Hortic. Sci. 13, 705–706. doi: 10.21273/HORTSCI.13.6.705

[B68] Miao M. Xu X. Chen X. Xue L. Cao B. (2007). Cucumber carbohydrate metabolism and translocation under chilling night temperature. J. Plant Physiol. 164, 621–628. doi: 10.1016/j.jplph.2006.02.005 16616970

[B69] Mlinarić S. Antunović Dunić J. Skendrović Babojelić M. Cesar V. Lepeduš H. (2017). Differential accumulation of photosynthetic proteins regulates diurnal photochemical adjustments of PSII in common fig (Ficus carica l.) leaves. J. Plant Physiol. 209, 1–10. doi: 10.1016/j.jplph.2016.12.002 27987432

[B70] Mlinarić S. Cesar V. Lepeduš H. (2021). Antioxidative response and photosynthetic regulatory mechanisms in common fig leaves after short-term chilling stress. Ann. Appl. Biol. 178, 315–327. doi: 10.1111/aab.12671

[B71] Moran R. Porath D. (1980). Chlorophyll determination in intact tissues using N,N-dimethylformamide. Plant Physiol. 65, 478–479. doi: 10.1104/pp.65.3.478 16661217 PMC440358

[B72] Murata N. Takahashi S. Nishiyama Y. Allakhverdiev S. I. (2007). Photoinhibition of photosystem II under environmental stress. Biochim. Biophys. Acta 1767, 414–421. doi: 10.1016/j.bbabio.2006.11.019 17207454

[B73] Nägele T. Kandel B. A. Frana S. Meißner M. Heyer A. G. (2011). A systems biology approach for the analysis of carbohydrate dynamics during acclimation to low temperature in arabidopsis thaliana. FEBS J. 278, 506–518. doi: 10.1111/j.1742-4658.2010.07971.x 21166998

[B74] Nievola C. C. Carvalho C. P. Carvalho V. Rodrigues E. (2017). Rapid responses of plants to temperature changes. Temperature 4, 371–405. doi: 10.1080/23328940.2017.1377812 PMC5800372 29435478

[B75] Oliveira G. Peñuelas J. (2005). Effects of winter cold stress on photosynthesis and photochemical efficiency of PSII of the Mediterranean cistus albidus l. and quercus ilex l. Plant Ecol. 175, 179–191. doi: 10.1007/s11258-005-4876-x

[B76] Osmond C. B. (1978). Crassulacean acid metabolism: A curiosity in context. Annu. Rev. Plant Physiol. 29, 379–414. doi: 10.1146/annurev.pp.29.060178.002115

[B77] Peeler T. C. Naylor A. W. (1988). A comparison of the effects of chilling on thylakoid electron transfer in pea (Pisum sativum l.) and cucumber (Cucumis sativus l.) 1. Plant Physiol. 86, 147–151. doi: 10.1104/pp.86.1.147 16665857 PMC1054445

[B78] Porra R. J. Thompson W. A. Kriedemann P. E. (1989). Determination of accurate extinction coefficients and simultaneous equations for assaying chlorophylls a and b extracted with four different solvents: verification of the concentration of chlorophyll standards by atomic absorption spectroscopy. Biochim. Biophys. Acta (BBA) - Bioenergetics 975, 384–394. doi: 10.1016/S0005-2728(89)80347-0

[B79] Rapacz M. (2007). Chlorophyll a fluorescence transient during freezing and recovery in winter wheat. Photosynthetica 45, 409–418. doi: 10.1007/s11099-007-0069-2

[B80] Ruelland E. Vaultier M.-N. Zachowski A. Hurry V. (2009). “Cold signalling and cold acclimation in plants,” in Advances in botanical research (Cambridge, Massachusetts: Academic Press), 35–150. doi: 10.1016/S0065-2296(08)00602-2

[B81] Sasaki H. Ichimura K. Imada S. Yamaki S. (2001). Sucrose synthase and sucrose phosphate synthase, but not acid invertase, are regulated by cold acclimation and deacclimation in cabbage seedlings. J. Plant Physiol. 158, 847–852. doi: 10.1078/0176-1617-00391

[B82] Seki M. Kamei A. Yamaguchi-Shinozaki K. Shinozaki K. (2003). Molecular responses to drought, salinity and frost: common and different paths for plant protection. Curr. Opin. Biotechnol. 14, 194–199. doi: 10.1016/S0958-1669(03)00030-2 12732320

[B83] Shikanai T. (2007). Cyclic electron transport around photosystem I: Genetic approaches. Annu. Rev. Plant Biol. 58, 199–217. doi: 10.1146/annurev.arplant.58.091406.110525 17201689

[B84] Srivastava A. Guissé B. Greppin H. Strasser R. J. (1997). Regulation of antenna structure and electron transport in photosystem II of pisum sativum under elevated temperature probed by the fast polyphasic chlorophyll a fluorescence transient: OKJIP. Biochim. Biophys. Acta (BBA) - Bioenergetics 1320, 95–106. doi: 10.1016/S0005-2728(97)00017-0

[B85] Srivastava A. Strasser R. J. Govindjee (1999). Greening of peas: Parallel measurements of 77 K emission spectra, OJIP chlorophyll a fluorescence transient, period four oscillation of the initial fluorescence level, delayed light emission, and P700. Photosynthetica 37, 365. doi: 10.1023/A:1007199408689

[B86] Stirbet A. (2013). Excitonic connectivity between photosystem II units: what is it, and how to measure it? Photosynth Res. 116, 189–214. doi: 10.1007/s11120-013-9863-9 23794168

[B87] Stirbet A. Govindjee (2011). On the relation between the kautsky effect (chlorophyll a fluorescence induction) and photosystem II: Basics and applications of the OJIP fluorescence transient. J. Photochem. Photobiol. B: Biol. 104, 236–257. doi: 10.1016/j.jphotobiol.2010.12.010 21295993

[B88] Strasser B. J. (1997). Donor side capacity of photosystem II probed by chlorophyll a fluorescence transients. Photosynthesis Res. 52, 147–155. doi: 10.1023/A:1005896029778

[B89] Strasser R. J. Srivastava A. Govindjee G. (1995). Polyphasic chlorophyll a fluorescence transient in plants and cyanobacteria*. Photochem. Photobiol. 61, 32–42. doi: 10.1111/j.1751-1097.1995.tb09240.x

[B90] Strasser R. J. Srivastava A. Tsimilli-Michael M. (2000). “The fluorescence transient as a tool to characterize and screen photosynthetic samples,” in Probing photosynthesis: Mechanisms regulation and adaptation. Eds. Yunus M. Pathre U. Mohanty P. (London: Taylor and Francis), 445–483.

[B91] Strasser B. J. Strasser R. J. (1995). “Measuring fast fluorescence transients to address environmental questions: The JIP-test,” in Photosynthesis: From light to biosphere. Ed. Mathis P. (Dordrecht: Kluwer Academic Publishers), 977–980.

[B92] Strasser R. J. Tsimilli-Michael M. Qiang S. Goltsev V. (2010). Simultaneous *in vivo* recording of prompt and delayed fluorescence and 820-nm reflection changes during drying and after rehydration of the resurrection plant haberlea rhodopensis. Biochim. Biophys. Acta (BBA) - Bioenergetics 1797, 1313–1326. doi: 10.1016/j.bbabio.2010.03.008 20226756

[B93] Strasser R. J. Tsimilli-Michael M. Srivastava A. (2004). “Analysis of the chlorophyll a fluorescence transient,” in Chlorophyll a fluorescence: A signature of photosynthesis advances in photosynthesis and respiration. Eds. Papageorgiou G. C. Govindjee (Dordrecht: Springer Netherlands), 321–362. doi: 10.1007/978-1-4020-3218-9_12

[B94] Suorsa M. Järvi S. Grieco M. Nurmi M. Pietrzykowska M. Rantala M.. (2012). PROTON GRADIENT REGULATION5 is essential for proper acclimation of arabidopsis photosystem I to naturally and artificially fluctuating light conditions. Plant Cell 24, 2934–2948. doi: 10.1105/tpc.112.097162 22822205 PMC3426124

[B95] Takahashi S. Murata N. (2006). Glycerate-3-phosphate, produced by CO2 fixation in the Calvin cycle, is critical for the synthesis of the D1 protein of photosystem II. Biochim. Biophys. Acta (BBA) - Bioenergetics 1757, 198–205. doi: 10.1016/j.bbabio.2006.02.002 16551463

[B96] Takahashi S. Murata N. (2008). How do environmental stresses accelerate photoinhibition? Trends Plant Sci. 13, 178–182. doi: 10.1016/j.tplants.2008.01.005 18328775

[B97] Tarkowski Ł.P. Van de Poel B. Höfte M. Van den Ende W. (2019). Sweet immunity: Inulin boosts resistance of lettuce (Lactuca sativa) against grey mold (Botrytis cinerea) in an ethylene-dependent manner. Int. J. Mol. Sci. 20, 1052. doi: 10.3390/ijms20051052 30823420 PMC6429215

[B98] Tay S. He J. Yam T. W. (2019). CAM plasticity in epiphytic tropical orchid species responding to environmental stress. Bot. Stud. 60, 7. doi: 10.1186/s40529-019-0255-0 31087187 PMC6513927

[B99] Terashima I. Huang L.-K. Osmond C. B. (1989). Effects of leaf chilling on thylakoid functions, measured at room temperature, in cucumis sativus l. and oryza sativa l. Plant Cell Physiol. 30, 841–850. doi: 10.1093/oxfordjournals.pcp.a077814

[B100] Terashima I. Kashino Y. Katoh S. (1991a). Exposure of leaves of cucumis sativus l. @ to low temperatures in the light causes uncoupling of thylakoids i. studies with isolated thylakoids. Plant Cell Physiol. 32, 1267–1274. doi: 10.1093/oxfordjournals.pcp.a078205

[B101] Terashima I. Sonoike K. Kawazu T. Katoh S. (1991b). Exposure of leaves of cucumis sativus l. @ to low temperatures in the light causes uncoupling of thylakoids II. non-destructive measurements with intact leaves. Plant Cell Physiol. 32, 1275–1283. doi: 10.1093/oxfordjournals.pcp.a078206

[B102] Tikkanen M. Aro E.-M. (2014). Integrative regulatory network of plant thylakoid energy transduction. Trends Plant Sci. 19, 10–17. doi: 10.1016/j.tplants.2013.09.003 24120261

[B103] Tikkanen M. Rantala S. Aro E.-M. (2015). Electron flow from PSII to PSI under high light is controlled by PGR5 but not by PSBS. Front. Plant Sci. 6. doi: 10.3389/fpls.2015.00521 PMC4495676 26217370

[B104] Toki T. Ogiwara S. Aoki H. (1978). Effect of varying night temperature on the growth and yields in cucumber. Acta Hortic. 87, 233–238. doi: 10.17660/ActaHortic.1978.87.24

[B105] Venkatesh J. Upadhyaya C. P. Yu J.-W. Hemavathi A. Kim D. H. Strasser R. J.. (2012). Chlorophyll a fluorescence transient analysis of transgenic potato overexpressing d-galacturonic acid reductase gene for salinity stress tolerance. Hortic. Environ. Biotechnol. 53, 320–328. doi: 10.1007/s13580-012-0035-1

[B106] Verspreet J. Cimini S. Vergauwen R. Dornez E. Locato V. Le Roy K.. (2013). Fructan metabolism in developing wheat (Triticum aestivum l.) kernels. Plant Cell Physiol. 54, 2047–2057. doi: 10.1093/pcp/pct144 24104051

[B107] Wattana P. (2003)Raffinose family oligosaccharide metabolism in crassulacean acid metabolism species *Plectranthus hadiensis* l (Accessed June 22, 2022).

[B108] Wellburn A. R. (1994). The spectral determination of chlorophylls a and b, as well as total carotenoids, using various solvents with spectrophotometers of different resolution. J. Plant Physiol. 144, 307–313. doi: 10.1016/S0176-1617(11)81192-2

[B109] Wilkinson S. Clephan A. L. Davies W. J. (2001). Rapid low temperature-induced stomatal closure occurs in cold-tolerant commelina communis leaves but not in cold-sensitive tobacco leaves, *via* a mechanism that involves apoplastic calcium but not abscisic acid. Plant Physiol. 126, 1566–1578. doi: 10.1104/pp.126.4.1566 11500555 PMC117156

[B110] Winter K. (2019). Ecophysiology of constitutive and facultative CAM photosynthesis. J. Exp. Bot. 70, 6495–6508. doi: 10.1093/jxb/erz002 30810162

[B111] Yamori W. Hikosaka K. Way D. A. (2014). Temperature response of photosynthesis in C3, C4, and CAM plants: temperature acclimation and temperature adaptation. Photosynth Res. 119, 101–117. doi: 10.1007/s11120-013-9874-6 23801171

[B112] Yamori W. Noguchi K. Terashima I. (2005). Temperature acclimation of photosynthesis in spinach leaves: analyses of photosynthetic components and temperature dependencies of photosynthetic partial reactions. Plant Cell Environ. 28, 536–547. doi: 10.1111/j.1365-3040.2004.01299.x

[B113] Yamori W. Sakata N. Suzuki Y. Shikanai T. Makino A. (2011). Cyclic electron flow around photosystem I *via* chloroplast NAD(P)H dehydrogenase (NDH) complex performs a significant physiological role during photosynthesis and plant growth at low temperature in rice. Plant J. 68, 966–976. doi: 10.1111/j.1365-313X.2011.04747.x 21848656

[B114] Yang X. Cushman J. C. Borland A. M. Edwards E. J. Wullschleger S. D. Tuskan G. A.. (2015). A roadmap for research on crassulacean acid metabolism (CAM) to enhance sustainable food and bioenergy production in a hotter, drier world. New Phytol. 207, 491–504. doi: 10.1111/nph.13393 26153373

[B115] Yang Y.-J. Zhang S.-B. Huang W. (2018). Chloroplastic ATP synthase alleviates photoinhibition of photosystem I in tobacco illuminated at chilling temperature. Front. Plant Sci. 9. doi: 10.3389/fpls.2018.01648 PMC6246715 30487806

[B116] Yusuf M. A. Kumar D. Rajwanshi R. Strasser R. J. Tsimilli-Michael M. Govindjee. (2010). Overexpression of γ-tocopherol methyl transferase gene in transgenic brassica juncea plants alleviates abiotic stress: Physiological and chlorophyll a fluorescence measurements. Biochim. Biophys. Acta (BBA) - Bioenergetics 1797, 1428–1438. doi: 10.1016/j.bbabio.2010.02.002 20144585

[B117] Zandalinas S. I. Fritschi F. B. Mittler R. (2021). Global warming, climate change, and environmental pollution: Recipe for a multifactorial stress combination disaster. Trends Plant Sci. 26, 588–599. doi: 10.1016/j.tplants.2021.02.011 33745784

[B118] Zhang Y. H. Chen L. J. He J. L. Qian L. S. Wu L. Q. Wang R. F. (2010). Characteristics of chlorophyll fluorescence and antioxidative system in super-hybrid rice and its parental cultivars under chilling stress. Biol. Plant 54, 164–168. doi: 10.1007/s10535-010-0027-x

[B119] Zhang N. Portis A. R. (1999). Mechanism of light regulation of rubisco: A specific role for the larger rubisco activase isoform involving reductive activation by thioredoxin-f. Proc. Natl. Acad. Sci. 96, 9438–9443. doi: 10.1073/pnas.96.16.9438 10430961 PMC17801

[B120] Zivcak M. Brestic M. Kalaji H. M. Govindjee (2014). Photosynthetic responses of sun- and shade-grown barley leaves to high light: is the lower PSII connectivity in shade leaves associated with protection against excess of light? Photosynth Res. 119, 339–354. doi: 10.1007/s11120-014-9969-8 24445618 PMC3923118

[B121] Živčák M. Brestič M. Olšovská K. Slamka P. (2008). Performance index as a sensitive indicator of water stress in *Triticum aestivum* L. Plant Soil Environ. 54, 133–139. doi: 10.17221/392-PSE

[B122] Żurek G. Rybka K. Pogrzeba M. Krzyżak J. Prokopiuk K. (2014). Chlorophyll a fluorescence in evaluation of the effect of heavy metal soil contamination on perennial grasses. PloS One 9, e91475. doi: 10.1371/journal.pone.0091475 24633293 PMC3954697

